# Morphologic classification of tracheobronchial arborization in children with congenital tracheobronchial stenosis and the associated cardiovascular defects

**DOI:** 10.3389/fped.2023.1123237

**Published:** 2023-05-23

**Authors:** Jie Hu, Hao Wang, Xinwei Du, Limin Zhu, Shunmin Wang, Haibo Zhang, Zhiwei Xu, Hao Chen

**Affiliations:** ^1^ Department of Cardiothoracic Surgery, Shanghai Children’s Medical Center, School of Medicine, Shanghai Jiao Tong University, Shanghai, China; ^2^ Department of Pediatric Cardiology, Xinhua Hospital, Shanghai Jiao Tong University School of Medicine, Shanghai, China

**Keywords:** congenital, tracheal stenosis, classification, children, cardiovascular anomalies

## Abstract

**Background:**

We sought to classify patients with congenital tracheal stenosis (CTS) according to tracheobronchial morphology and determine anatomic features associated with tracheobronchial anomalies (TBAs) and concurrent cardiovascular defects (CVDs).

**Methods:**

We enrolled 254 patients who underwent tracheoplasty between November 1, 2009 and December 30, 2018. The anatomic features of the tracheobronchial tree and cardiovascular system were abstracted from bronchoscopy, echocardiography, computerized tomography, and operative reports.

**Results:**

Four types of tracheobronchial morphology were identified: Type-1, which included normal tracheobronchial arborization (Type-1A, *n* = 29) and tracheal bronchus (Type-1B, *n* = 22); Type-2 (tracheal trifurcation; *n* = 49), and Type-3 (typical bridging bronchus; *n* = 47). Type-4 (bronchus with an untypical bridging pattern) was divided into Type-4A (involving bronchial diverticulum; *n* = 52) and Type-4B (absent bronchus; *n* = 55). Carinal compression and tracheomalacia were significantly more frequent in Type-4 patients than in the other patients (*P *< 0.01). CVDs were common in patients with CTS, especially in patients with Type-3 and Type-4 (*P *< 0.01). Persistent left superior vena cava was most common among patients with Type-3 (*P *< 0.01), and pulmonary artery sling was most frequent among those with Type-4 (*P *< 0.01). Outflow tract defects were most likely to occur in Type-1B. Early mortality was detected in 12.2% of all patients, and young age (*P *= 0.02), operation in the early era (*P *< 0.01), and bronchial stenosis (*P *= 0.03) were proven to be risk factors.

**Conclusions:**

We demonstrated a useful morphological classification for CTS. Bridging bronchus was most closely linked with vascular anomalies, while tracheal bronchus was frequently associated with outflow tract defects. These results may provide a clue to CTS pathogenesis.

## Introduction

Congenital tracheal stenosis (CTS) is a rare but potentially life-threatening disease in children. The clinical presentation can vary from almost asymptomatic to recurrent dyspnea, stridor, or even near-fatal airway obstruction. Most children with CTS are characterized by complete cartilaginous rings and are usually associated with various congenital malformations, particularly cardiovascular defects (CVDs) and tracheobronchial anomalies (TBAs) (1–3). Although slide tracheoplasty has achieved satisfactory airway reconstruction results, the overall clinical outcomes remain strongly influenced by concurrent tracheobronchial and cardiovascular malformations (4–7). Thus, it is essential to identify these anomalies prior to surgical intervention, especially concurrent cardiac surgery and tracheoplasty, to avoid complications.

In our experience, several CVDs, including pulmonary artery sling (PAS), ventricular or atrial septal defects, and tetralogy of Fallot or pulmonary atresia, are frequently associated with CTS. The association of PAS and CTS is often referred to as a “ring–sling complex” (RSC) owing to the prevalence of complete cartilaginous rings (8–10). However, the relationship between cardiovascular defects and specific tracheobronchial morphologies in children with CTS has not been thoroughly explored.

To the best of our knowledge, various abnormalities of the tracheobronchial tree have been identified using postmortem specimens and radiological findings. In the Wells classification, CTS is commonly associated with tracheal bronchus (TB) and bridging bronchus (BB) (9, 11). In the descriptions of Great Ormond Street Hospital, tracheal trifurcation was identified normally, and the extension of the stenosis into one or both bronchi was also common (12). Currently, there are no uniform classification systems that comprehensively describe the numerous tracheobronchial morphologies in children with CTS. Therefore, we proposed the addition of two new subgroups to the four CTS categories formulated by Wells and colleagues. The objective of this study was to describe different tracheobronchial patterns and various cardiovascular anomalies in patients with CTS and determine whether an association exists between TBAs and concurrent CVDs.

## Methods

### Patients

We conducted an observational, single-center, retrospective study. All 254 patients who underwent surgical repair for CTS between November 1, 2009 and December 30, 2018 at Shanghai Children's Medical Center were included. Data were obtained by reviewing medical records from admission until the first follow-up at 1 month after discharge. This study was approved by the hospital's ethics committee (SCMCIRB-W2022008), and the requirement of informed consent was waived. To compare mortality rates throughout the study period, the surgical era was classified into two timeframes: 2008–2014 and 2015–2018, according to the operative quantity.

### Tracheobronchial morphology

**Figure 1:**
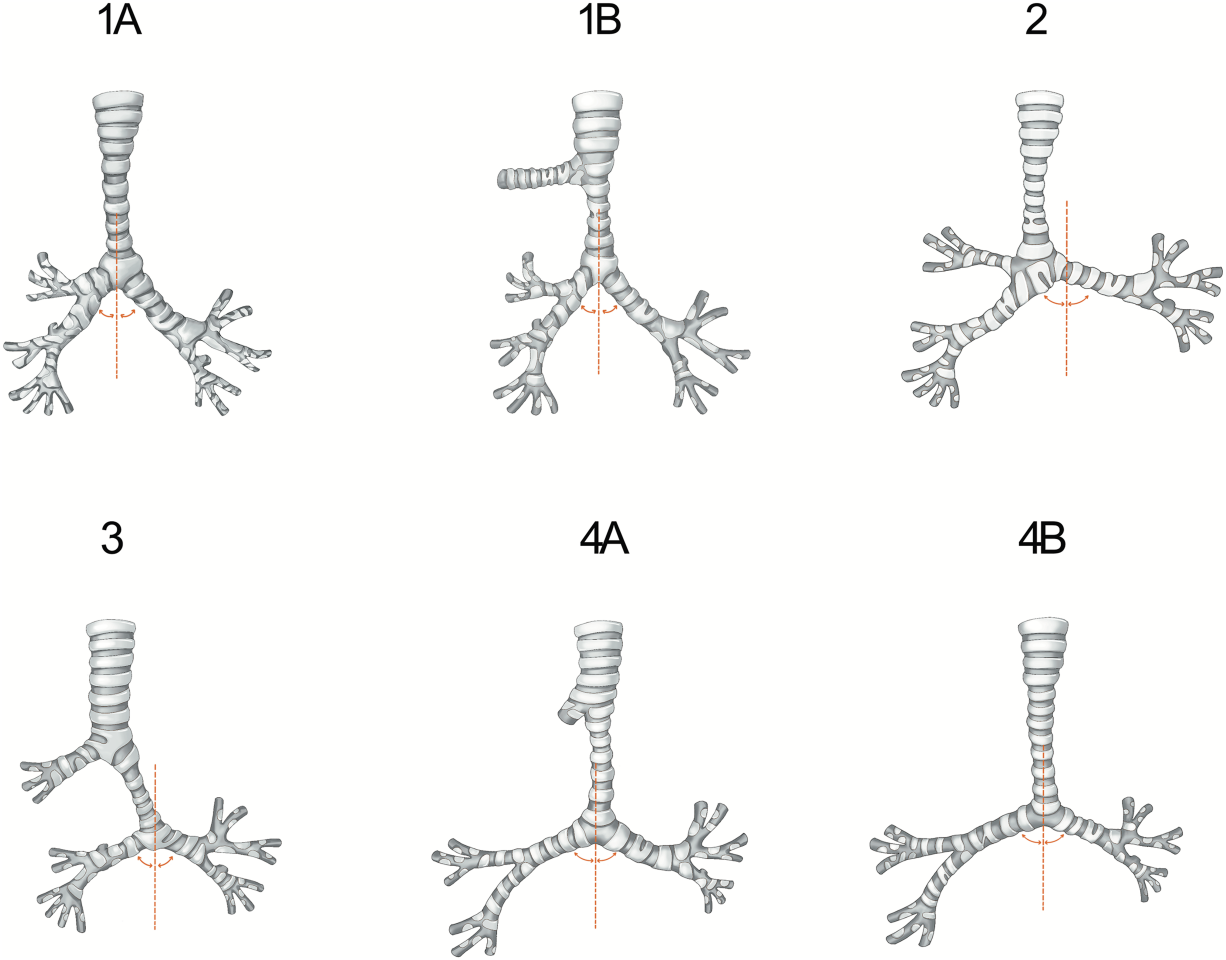
This figure illustrates the types of tracheobronchial arborization included in our study. Type-1A refers to a normal tracheobronchial pattern. In Type-1B, the right upper lobe is supplied by a right tracheal bronchus. In Type-2, the trachea directly divides into three bronchi. The right lower lobe is supplied by a typical bridging bronchus in Type-3, and the right upper lobe bronchus is present only as a short diverticulum in Type-4A or absent in Type-4B. The curved lines represent the right and left bronchial angles in relation to the midline.

Airway anatomy was assessed and defined by a multidisciplinary tracheal team, based on a combination of bronchoscopy, computed tomography (CT), and intraoperative findings. Tracheobronchial arborization types include (9, 13) Type-1A [normal tracheobronchial arborization in which the trachea divides at the carina into the left and right main bronchi, and the right upper lobe bronchus (ULB) originates from the right main bronchus], Type-1B (TB; which is described as a right ULB originating from the right side of the trachea, above the carina), Type-2 (tracheal trifurcation; which involves an ectopic right ULB directly arising at the level of the carina), Type-3 (typical BB; which is a malformation in which the right main bronchus ends in the right ULB and the right intermediate bronchus abnormally arises from the left main bronchus, forming a pseudocarina), and Type-4 (atypical BB; which is similar to Type-3, but the right main bronchus is present only as a short “bronchial diverticulum” [Type-4A] or is absent [Type-4B], and the lower bifurcation (pseudocarina) shows a typical inverted “T” pattern). The specific airway anatomical types are shown in Figure 1.

Bronchial stenosis was deﬁned as the presence of complete tracheal rings in at least one bronchus beyond the carina, with greater than 30% narrowing. Carinal compression was deﬁned as focal narrowing (>30% decrease in cross-sectional area) at the level of the carina. Tracheomalacia was defined as a dynamic collapse of the trachea that exceeded 50% of the diameter on expiration, as seen on bronchoscopy.

### Surgical management

All patients had symptoms of tracheal compression, ranging from mild stridor to severe respiratory distress. Diagnosis of CVD was routinely confirmed by a review of echocardiographic and CT reports before the operation. The surgical procedure was performed through a median sternotomy, with cardiopulmonary bypass. Where necessary, intracardiac repair was performed first and PAS repair was completed subsequently. Tracheal repair techniques included slide tracheoplasty, patch tracheoplasty (pericardial patch and tracheal autograft patch repair), and resection with end-to-end anastomosis (14). Early mortality was defined as death occurring within 30 days of surgery or prior to hospital discharge.

### Statistical analysis

Continuous variables are presented as median with interquartile range for skewed variables. Continuous data were compared using the Kruskal–Wallis test. Categorical variables are described as numbers with percentages and were compared using the *χ*^2^ test. Fisher's exact test was applied when the expected frequency was less than 5. Univariate and multivariate logistic regression analyses were performed to identify possible risk factors for early mortality. All analyses were performed using SPSS statistical software version 25 (SPSS Inc., Chicago, USA).

## Results

### Baseline characteristics and tracheobronchial morphology

Baseline characteristics are presented in Table 1. Among the 254 patients with CTS, 51 (20.1%) patients had Type-1, including 29 with Type-1A and 22 with Type-1B; 49 (19.3%) patients had Type-2, 47 (18.5%) patients had Type-3, and 107 (42.1%) patients had Type-4 (52 with Type-4A and 55 with Type-4B). The prevalence of carinal compression (54/107, 50.5%) or tracheomalacia (58/107, 54.2%) was significantly higher in the Type-4 group than in the other groups (*P *< 0.01). Bronchial stenosis was present in 60 patients (23.6%). However, there was no significant difference between the four groups.

**Table 1:** Baseline characteristics of patient groups.

	Type-1 (*N* = 51)	Type-2 (*N* = 49)	Type-3 (*N* = 47)	Type-4 (*N* = 107)	*P*-value
Sex (Male)	34 (66.7%)	34 (69.4%)	20 (42.6%)	62 (57.9%)	0.03
Weight (kg)	9.4 (7.3; 11.0)	9.0 (8.2; 11.5)	9.0 (7.6; 10.9)	9.0 (7.8; 11.0)	0.48
Age (year)	1.3 (0.9; 2.4)	1.30 (1.0; 2.1)	1.2 (1.0; 1.7)	1.2 (1.0; 1.7)	0.50
Cardiovascular defects	42 (82.4%)	43 (87.8%)	46 (97.9%)	103 (96.3%)	<0.01
Extra-cardiovascular defects	5 (9.8%)	5 (10.2%)	5 (10.6%)	5 (4.7%)	0.45
Cardiac malposition	2 (3.9%)	6 (12.2%)	4 (8.5%)	10 (9.3%)	0.20
Pulmonary hypoplasia	0 (0.0%)	5 (10.2%)	2 (4.3%)	5 (4.7%)	0.11
Bronchial stenosis	7 (13.7%)	15 (30.6%)	10 (21.3%)	28 (26.2%)	0.20
Carinal compression	9 (17.6%)	8 (16.3%)	12 (25.5%)	54 (50.5%)	<0.01
Tracheomalacia	14 (27.5%)	10 (20.4%)	22 (46.8%)	58 (54.2%)	<0.01

Among the four CTS groups, the Type-3 group had a lower proportion of male (20/47, 42.6%) than the other three groups (*P *= 0.03). CVDs were common in all groups and were most predominant in the Type-3 (46/47, 97.9%) and Type-4 (103/107, 96.3%) (*P *< 0.01) groups. There were various types of extracardiac defects in 20 (7.9%) patients, but no significant difference was found between the four groups. Similarly, there was no significant difference between the four groups in the prevalence of cardiac malposition and pulmonary hypoplasia.

### Association between TBAs and CVDs

Two hundred and thirty-four patients (92.1%) had various types of cardiovascular malformations (Table 2). The major types of CVDs were PAS (*n* = 168, 66.1%), persistent left superior vena cava (PLSVC) (*n* = 82, 32.3%), atrial septal defect (*n* = 69, 27.2%), patent ductus arteriosus (*n* = 39, 15.4%), and ventricular septal defect (VSD) (*n* = 36, 14.2%). Right/double aortic arch (*n* = 18, 7.1%) and tetralogy of Fallot (*n* = 15, 5.9%) were also common in the entire cohort. However, left heart obstructive lesions (*n* = 3, 1.2%), including mitral stenosis, aortic stenosis, and coarctation of the aorta, were rare among all patients.

**Table 2:** Relationship between different tracheobronchial anomalies and cardiovascular defects.

	Type-1 (*N* = 51)	Type-2 (*N* = 49)	Type-3 (*N* = 47)	Type-4 (*N* = 107)	*P*-value
PLSVC	13 (25.5%)	9 (18.4%)	25 (53.2%)	35 (32.7%)	<0.01
PAS	7 (13.7%)	34 (69.4%)	32 (68.1%)	95 (88.8%)	<0.01
DAA/RAA	7 (13.7%)	4 (8.2%)	3 (6.4%)	4 (3.7%)	0.14
VSD	16 (31.4%)	0 (0.0%)	10 (21.3%)	10 (9.4%)	<0.01
ASD	13 (25.5%)	12 (24.5%)	19 (40.4%)	25 (23.4%)	0.16
PDA	9 (17.6%)	8 (16.3%)	6 (12.8%)	16 (15.0%)	0.92
PAVC	0 (0.0%)	1 (2.0%)	1 (2.1%)	0 (0.0%)	0.18
APVR	3 (5.9%)	0 (0.0%)	2 (4.3%)	0 (0.0%)	0.02
PS/Sub-PS	3 (5.9%)	0 (0.0%)	1 (2.1%)	1 (0.9%)	0.15
CTD	9 (17.6%)	4 (8.2%)	7 (14.9%)	6 (5.6%)	0.07
TOF	5 (9.8%)	2 (4.1%)	4 (8.5%)	4 (3.7%)	0.34
PA-VSD	1 (2.0%)	2 (4.1%)	0 (0.0%)	1 (0.9%)	0.39
DORV	3 (5.9%)	0 (0.0%)	3 (6.4%)	1 (0.9%)	0.05
LHOL	1 (2.0%)	0 (0.0%)	1 (2.1%)	1 (0.9%)	0.59
MS	1 (2.0%)	0 (0.0%)	0 (0.0%)	0 (0.0%)	0.58
AS	0 (0.0%)	0 (0.0%)	0 (0.0%)	1 (0.9%)	1.00
CoA	0 (0.0%)	0 (0.0%)	1 (2.1%)	0 (0.0%)	0.19

APVR, anomalous pulmonary venous return; AS, aortic stenosis; ASD, atrial septal defect; CoA, coarctation of aorta; CTD, conotruncal defects; DAA, double aortic arch; DORV, double outlet right ventricle; LHOL, left heart obstructive lesions; MS, mitral stenosis; PAS, pulmonary artery sling; PAVC, partial atrioventricular canal; PA-VSD, pulmonary atresia with ventricular septal defect; PDA, patent ductus arteriosus; PLSVC, persistent left superior vena cava; PS, pulmonary stenosis; RAA, right aortic arch; TOF, tetralogy of Fallot; VSD, ventricular septal defect.

We analyzed the major concurrent cardiovascular anomalies that could be associated with different tracheobronchial subtypes. Overall, CVDs were more frequent in patients with BB anomaly (Type-3 and 4, *P *< 0.01). PLSVC was common in all subtypes of CTS but was significantly correlated with Type-3 (*P *< 0.01). Compared to Type-1A and 1B, PAS was more frequent in the other four subtypes and was markedly associated with Type-4A and 4B (*P *< 0.01). VSD was most correlated with the presentation of Type-1B (*P *< 0.01). When combined with conotruncal defects, the correlation between these two types of outflow tract defects and Type-1B was further strengthened. However, outflow tract defects were uncommon in Type-2 and 4B. Type-2 was not significantly correlated with the main types of CVDs, excluding PAS.

### Early mortality and postoperative hospital stay

The distribution of patients according to CTS type, operative era, and early outcomes are outlined in Table 3. There were 31 (12.2%) early deaths, with 6 (11.8%), 6 (12.2%), and 19 (17.8%) cases in the Type-1, 2, and 4 groups, respectively. No early deaths (0%, 0/47) occurred among Type-3 patients. Early mortality was significantly higher in patients with atypical BB (Type-4, *P *= 0.02). In univariate analysis, age at operation, surgical technique, operative era, carinal compression, and tracheomalacia were predictors of early mortality (for all comparisons, *P *< 0.05) (Supplementary Table S1). Furthermore, young age [odds ratio (OR) = 1.00, *P *= 0.02], operation in the early era (OR = 4.10, *P *< 0.01), and bronchial stenosis (OR = 2.80, *P *= 0.03) were proven to be risk factors for early death in the multivariate model (Supplementary Table S2).

**Table 3:** Operative and postoperative outcomes.

	Type-1 (*N* = 51)	Type-2 (*N* = 49)	Type-3 (*N* = 47)	Type-4 (*N* = 107)	*P*-value
Tracheoplasty					<0.01
Slide	38 (74.5%)	47 (95.9%)	29 (61.7%)	97 (90.7%)	
Patch	1 (2.0%)	1 (2.0%)	1 (2.1%)	4 (3.7%)	
End-to-End	12 (23.5%)	1 (2.0%)	17 (36.2%)	6 (5.6%)	
Operative era					0.04
2009–2014	24 (47.1%)	17 (34.7%)	23 (48.9%)	31 (29.0%)	
2015–2018	27 (52.9%)	32 (65.3%)	24 (51.1%)	76 (71.0%)	
Length of stay (day)	14.0 (11.0; 21.0)	15.0 (13.0; 22.0)	14.0 (12.5; 19.5)	17.0 (14.0; 26.5)	0.03
Early death	6 (11.8%)	6 (12.2%)	0 (0.00%)	19 (17.8%)	0.02

The median postoperative length of hospital stay was 15 days [interquartile range (IQR), 13–24 days]. Following tracheoplasty, children with atypical BB anomaly (Type-4) had longer length of hospital stay (17 days; IQR, 14–27 days) compared to the other patients (*P *= 0.03). Preoperative variables that were significantly associated with postoperative length of hospital stay in univariate analysis included carinal compression (*P *< 0.01), tracheomalacia (*P *< 0.01), concurrent CVDs (*P *= 0.03), and weight at surgery (*P *< 0.01) (Supplementary Table S3). Nonetheless, no risk factors were associated with postoperative length of hospital stay in the multivariate analysis.

## Discussion

Until recently, descriptions of CTS have been primarily limited to rare cases and various tracheobronchial anomalies. Great Ormond Street Hospital and Wells classifications have made good attempts at differentiating morphologic variants of both bronchial arborization and stenotic segments (9, 11, 12). However, neither of these classification systems adequately describe the airway morphological features that are currently associated with CTS patients. Our cohort is unique in that it is the largest single-institutional study of CTS cases. In our experience, all tracheobronchial morphologies could be identified by routine chest CT and classified into four main types and six subtypes. BB is the most common tracheobronchial positional anomaly and is most frequently associated with tracheobronchial malacia and carinal compression. In addition, PLSVC and PAS, which are two typical concurrent vascular malformations, tend to be more frequent in individuals with BB anomalies. Contrarily, VSD and CTD, which are the most common outflow tract defects, have no significant relationship with BB anomalies, especially untypical subtypes.

### Bridging bronchus

CTS patients with tracheobronchial abnormalities accounted for approximately 80% of our study population, and the surgeons had to devise various surgical techniques to repair complex forms of tracheal stenosis. In the whole population, the incidence of BB was 60.6% (Type-3 and 4 groups) and that of TB was only 8.7% (Type-1B group). In the cohort of 168 patients with RSC, BB tended to be more frequent (75.6%) than TB (2.4%). Notably, this disparity of coexisting malformation was more obvious in the Type-4 group (56.5% in RSC vs. 42.1% in CTS).

The high frequency of BB in patients with CTS, especially RSC, is a plausible clue to the underlying mechanism. Kishimoto and colleagues revealed that tracheal mesenchymal populations play pivotal roles in tracheal morphogenesis via mechanical characteristics (15). Developmental anomalies of these tissues may result in tracheobronchial malformations, such as tracheal stenosis, agenesis, and malacia. Gonzales-Crussi and colleagues proposed that interactions of ectopic mesenchyme (“exceptional conditions”) with the main bronchi may result in the formation of BB (16). Meanwhile, the development of the tracheobronchial tree is accompanied by parallel development of the pulmonary vasculature that originates from the sixth pair of aortic arches. Chen and colleagues hypothesized that widening of the peritracheal mesenchyme in CTS might provide a roomy space for the left postbranchial vessels to approach the right ventral sixth branchial arch, forming PAS (17). However, current theories do not account for the morphogenesis of BB after the formation of the main bronchi.

The tracheobronchial tree in Type-4 is an extremely rare bronchial malformation, with the right main bronchus present only as a short “diverticulum” in Type-4A or absent in Type-4B. There was no significant difference between these two subtypes in the concomitant intra- and extra-malformations and in early postoperative outcomes. In contrast, tracheomalacia and carinal compression were more common in Type-4B than in Type-4A (Table 1). In young children, Type-4B is occasionally confused with Type-1A when the pseudocarina remains at the T4-T5 level. The bronchial angle of Type-4B is increased and seems to have an inverted T appearance. Moreover, further bronchoscopy often reveals variations in the right bronchial distribution. These two important characteristics are critical in identifying the various subtypes (13).

**Figure 2:**
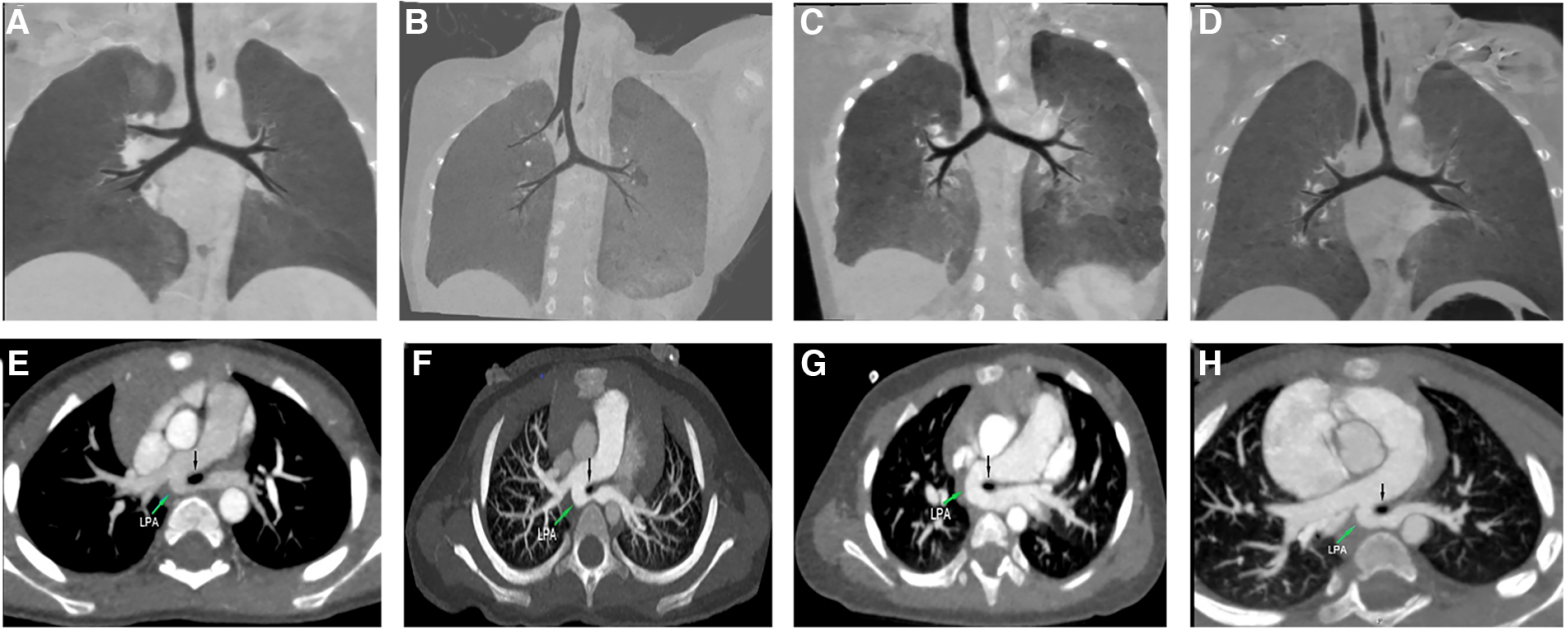
Examples of tracheobronchial branching abnormalities and concurrent pulmonary artery sling (PAS) on computed tomography. (**A**) Tracheal trifurcation in Type-2 group. (**B**) Typical bridging bronchus in Type-3 group. (**C**) The right main bronchus is present as a short “bronchial diverticulum” in Type-4A group. (**D**) The right main bronchus is absent in Type-4B group. (**C,D**) Are two subgroups of atypical bridging bronchus. (**E–H**) Axial images of the corresponding patients showing the left PAS arising from the right pulmonary artery. The PAS is seen compressing the stenotic trachea (black arrow).

In both tracheal trifurcation and bridging bronchus subtypes, which are closely related to PAS, the bronchial angle of the pseudocarina increased significantly (Figure 2). Such tracheobronchial features suggest that the aberrant left pulmonary artery, which runs posteriorly from the right pulmonary artery, may exert mechanical resistance to the adjacent tracheobronchial tree during embryonic development (16). Embryonically, the primary bronchial buds enlarge to form the right and left main bronchi, aerating two separate lungs. In contrast, the left pulmonary artery located on the right-posterior side of the trachea prevents the formation of the carina and the right ULB, causing an increase in the bronchial angle.

### Bronchial stenosis

Bronchial stenosis, as an extension of tracheal stenosis, is common and is present in almost 25% of the CTS population (4, 12, 18, 19). In our series, bronchial stenosis was noted in 23.6% of the population and was more common in the Type-2 (30.6%) and Type-4 (26.2%) groups, although the difference was not significant. The higher prevalence of bronchial stenosis in these specific morphologies implies that the stenosis may be due to the absence of contribution from the displaced or hypoplastic bronchi in fetal life (19).

Bronchial stenosis is speculated to be associated with a poor prognosis. In our experience with slide tracheoplasty, bronchial stenosis with concomitant carinal stenosis and compression was significantly associated with postoperative tracheomalacia and mortality (4). Our current study further demonstrates that the involvement of bronchial stenosis has an adverse effect on early postoperative outcomes. Coexisting bronchial stenosis reflects the severity of the underlying airway anatomical abnormality and is frequently associated with unilateral pulmonary hypoplasia. Cetrano and colleagues suggested that bronchial mismatches of greater than 20% can identify patients at increased risk for surgical reintervention and chronic respiratory failure (20). Bronchoplasty may be required in this group of patients and should be performed when possible.

### PLSVC and TBA

PLSVC, the most common venous anomaly, has been considered to be strongly associated with both intra- and extracardiac anomalies. Previous studies demonstrated a high incidence of intracardiac anomalies, including coarctation of the aorta, double-outlet right ventricle, and VSD, and the most common extracardiac anomaly was esophageal atresia (21, 22). Our study is the first to report a special association between PLSVC and TBAs. Besides, PLSVC was found to be more positively associated with an increased risk of BB, even when present as an isolated finding.

During the early embryonic stage, both the esophagus and respiratory tract are derived from the primitive foregut, and the association between digestive and respiratory malformations could be linked to a developmental disorder originating from the division of the primitive foregut into the lung bud and esophagus, which usually affects the right upper and middle lobes. The high prevalence of PLSVC in our series of patients with TBAs is similar to that reported in a series of patients with esophageal atresia (23), suggesting a common etiology. Future embryological studies may provide more evidence for this hypothetical common morphogenesis.

It is also speculated that the combination of PLSVC with intra- and extracardiac defects might be part of a continuum of abnormalities in the secondary heart field. Disorders in the development of this field have been found to be related to complex cardiac phenotypes and possibly to PLSVC (22–24). The correlation between PLSVC and BB anomalies is not surprising because the development of the secondary heart field was found to be closely related to that of the adjacent endoderm, which is the origin of many respiratory, gastrointestinal, and genitourinary organs, during the embryonic period.

### CVDs and TBAs

Similar to previous reports, patients with TBAs were commonly found to have concurrent CVDs in our study. The most common types were PAS and PLSVC, which are vascular anomalies. In all coexisting intracardiac anomalies, atrial septal defect, patent ductus arteriosus, and VSD were the major simple types, and conotruncal defects were the most prevalent type of the complex type. The association between CVDs, especially conotruncal defects and tracheobronchial branching anomalies, may involve common signaling pathways (25). For example, Shh–null mice may develop a phenocopy of pulmonary atresia, but they are also known to have bilateral hypoplastic lungs attributable to the absence of branching morphogenesis (26, 27).

Although arising from the endoderm, the development of the tracheobronchial epithelium is induced by epitheliomesenchymal interactions that require close proximity of the epithelium and mesenchyme (28). The position of the tracheal primordium is reportedly influenced by the adjacent cardiac tissue for ex vivo culture experiments, which showed that the foregut endoderm can differentiate into Nkx2-1^+^ respiratory epithelial progenitors in the presence of cardiac tissue (29). Moreover, variant transcription factors and signaling pathways, such as Nkx2-1, Sox2, Shh, Wnt, and bone morphogenetic proteins, play central roles in all dynamic morphogenetic processes (28–31). Sinner and colleagues found that mutations in genes within the Shh and Wnt pathways may lead to complete tracheal ring deformity (31). These transcription factors and signaling pathways are known to be involved in embryonic heart development, including the formation of the heart tube and the septation of the outflow tract.

### Limitations

This study has several limitations. First, because of the observational and retrospective design, there may have been missing data and selection bias. CTS is a rare malformation, and patients without obvious respiratory symptoms were not included in our morphological study of the tracheobronchial tree, especially children with normal bronchial branching. However, in our institution, patients with CTS are likely to undergo echocardiography before CT to evaluate the anatomical features of the cardiovascular system. This may be why patients with CVDs were highly represented in our cohort. Furthermore, CTS patients with unilateral bronchial and lung agenesis are extremely rare compared to those in previous studies (5, 12). Therefore, this special tracheobronchial morphology could not be statistically analyzed in our study. However, evaluating concurrent TBAs and CVDs in children with pulmonary malformations could be of great interest because this presentation may have very important interactions at the embryological origin. Another study limitation was the definition we used for bronchial stenosis, which included diagnosis using CT, because of the difficulty and danger associated with confirming severe tracheal obstruction via bronchoscopy (4). However, this important feature can be overestimated by CT, which was also noted in some cases in our series. Further investigations are needed to precisely evaluate the prevalence of bronchial stenosis and the influence of bronchial stenosis on early postoperative results.

## Conclusions

We proposed a morphologic classification for tracheobronchial arborization of CTS and demonstrated that this comprehensive classification is useful for describing congenital anomalies of the tracheobronchial tree. In particular, we observed a high frequency of bridging bronchi in patients with CTS. Our results suggest an interaction between various features of cardiovascular defects and different tracheobronchial branching morphologies and may provide a new clue for pathogenesis. Finally, because it is an important risk factor for early death, bronchial stenosis should be considered essential in making surgical prognosis.

## Data Availability

The original contributions presented in the study are included in the article/**Supplementary Material**, further inquiries can be directed to the corresponding author.
