# Supplementary material for: Morphologic classification of tracheobronchial arborization in children with congenital tracheobronchial stenosis and the associated cardiovascular defects
Source: Front Pediatr. 2023 May 23;11:1123237. doi: 10.3389/fped.2023.1123237 (PMC10242125; doi:10.3389/fped.2023.1123237)
Supplement: Supplementary file 1 [file Table1.pdf]

SUPPLEMENTAL TABLE 1. Univariate analysis for risk factors associated with early mortality

	No (n = 223)	Yes (n = 31)	Univariate analysis	
			OR (95% CI)	*P* value
**Male sex**	131 (58.7%)	19 (61.3%)	1.11 (0.51-2.40)	0.79
**Weight**	9.00 [8.00;11.0]	8.50 [7.20;11.8]	1.05 (0.98-1.13)	0.15
**Age**	1.20 [1.00;1.80]	1.30 [0.95;2.60]	1.00 (1.00-1.01)	0.04
**CTS types**				
**Type-1**	45 (20.2%)	6 (19.4%)	Ref	Ref
**Type-2**	43 (19.3%)	6 (19.4%)	1.05 (0.31-3.49)	0.94
**Type-3**	47 (21.1%)	0 (0.00%)	0.00 (0.00- Inf)	0.99
**Type-4**	88 (39.5%)	19 (61.3%)	1.62 (0.60-4.33)	0.34
**Tracheoplasty**				
**Patch**	4 (1.8%)	3 (9.7%)	Ref	Ref
**Slide**	184 (82.5%)_	27 (87.1%)	0.20 (0.04-0.92)	0.04
**End-to-end**	35 (15.7%)	1 (3.2%)	0.04 (0.00-0.46)	0.01
**Operative era**				
**2015-2018**	123 (55.2%)	19 (61.3%)	Ref	Ref
**2009-2014**	100 (44.8%)	23 (74.2%)	3.06 (1.41-6.64)	<0.01
**Extra-cardiovascular defects**	19 (8.52%)	1 (3.23%)	0.36 (0.05-2.77)	0.33
**Cardiovascular defects**	205 (91.9%)	29 (93.5%)	1.27 (0.28-5.77)	0.75
**Carinal compression**	65 (29.1%)	18 (58.1%)	3.37 (1.56-7.27)	<0.01
**Tracheomalacia**	83 (37.2%)	21 (67.7%)	3.54 (1.59-7.89)	<0.01
**Bronchial stenosis**	49 (22.0%)	11 (35.5%)	1.95 (0.88-4.35)	0.10
**Pulmonary dysplasia**	9 (4.04%)	3 (9.68%)	2.55 (0.65-9.97)	0.18

CTS, congenital tracheal stenosis; OR, odds ratio.

SUPPLEMENTAL TABLE 2. Multivariate analysis for risk factors associated with early mortality

	Early death	
	**OR (95% CI)**	***P* value**
**Weight**	0.87 (0.71-1.05)	0.15
**Age**	1.00 (1.00-1.01)	0.02
**Operative era**	4.10 (1.65-10.16)	<0.01
**Carinal compression**	2.38 (0.59-9.59)	0.22
**Tracheomalacia**	2.43 (0.58-10.16)	0.22
**Bronchial stenosis**	2.80 (1.10-7.10)	0.03
**Pulmonary dysplasia**	0.34 (0.07-1.75)	0.20

OR, odds ratio.

SUPPLEMENTAL TABLE 3. Univariate analysis for risk factors associated with postoperative length of stay

	***P* value**
**Male sex**	0.28
**Weight**	<0.01
**Age**	0.33
**CTS types**	0.16
**Tracheoplasty**	0.47
**Operative era**	0.31
**Extra-cardiovascular defects**	0.33
**Cardiovascular defects**	0.03
**Carinal compression**	<0.01
**Tracheomalacia**	<0.01
**Bronchial stenosis**	0.56
**Pulmonary dysplasia**	0.54

CTS, congenital tracheal stenosis.
